# U.S. National Park visitor perceptions and behavioral intentions towards actions to prevent white-nose syndrome

**DOI:** 10.1371/journal.pone.0278024

**Published:** 2022-11-23

**Authors:** Hannah G. Shapiro, Adam S. Willcox, Emma V. Willcox, Michelle L. Verant

**Affiliations:** 1 Warnell School of Forestry and Natural Resources, University of Georgia, Athens, Georgia, United States of America; 2 Department of Forestry, Wildlife and Fisheries, University of Tennessee, Knoxville, Tennessee, United States of America; 3 Smith Center for International Sustainable Agriculture, University of Tennessee, Knoxville, Tennessee, United States of America; 4 Biological Resources Division, National Park Service, Fort Collins, Colorado, United States of America; University of Oklahoma Norman Campus: The University of Oklahoma, UNITED STATES

## Abstract

In the United States, the discovery and spread of white-nose syndrome (WNS) has drastically changed how bats and caves are managed. The U.S. National Park Service has been instrumental in the national response to WNS, as it manages extensive cave resources and has a close relationship with the public. However, managers lack information on visitor support for disease prevention measures designed to slow the spread of WNS and minimize human disturbance of vulnerable bat populations. This study utilized the Theory of Planned Behavior to determine how visitor attitudes, subjective norms, and perceived behavioral controls influenced their behavior regarding WNS preventive actions, including participation in educational programming on bats, wearing clothes or shoes in caves that have not been contaminated with the fungus that causes WNS, walking over decontamination mats, and complying with cave closures. During summer of 2019, data were collected using an on-site survey of 1365 visitors to eight U.S. national park units: Oregon Caves, Lava Beds, Carlsbad Caverns, El Malpais, Wind Cave, Jewel Cave, Mammoth Cave, and Cumberland Gap. Visitors were willing to participate in all preventative actions addressed in the survey (77.7%-96.7%). Visitors expressed that engaging in these actions was very desirable (36.0%-65.6%), and their decision to engage in these actions was most strongly influenced by park staff (39.2%-68.8%) or signage (35.5%-61.9%). Attitudes and subjective norms were positive predictors of behavioral intentions for all measures. Perceived behavioral control was not a direct predictor for behavioral intent, but its interaction with attitudes and subjective norms had a moderating influence on intention to comply with multiple WNS preventive actions. With the continued spread of WNS and emergence of other threats to bats, understanding visitor behavioral intent and underlying factors will facilitate successful implementation of preventive actions that are publicly supported and promote conservation of bat populations in U.S. national parks.

## Introduction

Cave management has changed drastically over the past decade, as the arrival of white-nose syndrome (WNS) in North America created new considerations and priorities for how caves used by bats are managed. White-nose syndrome is a disease caused by the fungus *Pseudogymnoascus destructans* (*Pd*), which infiltrates the skin tissues of bats during hibernation resulting in dehydration, winter emergence and exposure, interrupted metabolic activities, starvation, and death [1–3]. White-nose syndrome has killed millions of bats since its discovery and has caused population declines of over 90% in northern long-eared (*Myotis septentrionalis*), little brown (*Myotis lucifugus*), and tri-colored bat (*Perimyotis* subflavus) populations in less than 10 years [4]. WNS has been confirmed in 12 bat species in 38 states, including two endangered species (Gray bat *Myotis griescens* and Indiana bat *Myotis sodalist*) and one threatened species (Northern long-eared bat *Myotis septentrionalis*). *Pd* has been found on six additional species (without the confirmation of the disease), including two endangered species (Virginia big-eared bat *Corynorhinus townsendii virginianus* and Ozark big-eared bat *Corynorhinus townsendii ingens*) [5]. To date, the spread of WNS has primarily been attributed to bat-to-bat or bat-to-cave-to-bat transmission; however, there is evidence the fungus can persist within cave environments for years and can be spread to different locations by humans on gear or clothing [6–10].

In response to the devastating effects of WNS on North American bats, a national interagency group developed recommendations for cave access and decontamination [11, 12] using the best available science and universal precautions [13] to reduce anthropogenic disturbance to bats and minimize the risk for human-mediated spread of *Pd* [14]. One of the organizations that has been actively involved in the national response to WNS is the U.S. National Park Service (NPS), as it manages extensive cave resources and has direct contact with the public. Parks across the country have implemented varying measures to slow the spread of WNS and protect bats, including providing educational materials on WNS, expanding bat and cave interpretive programs to include information on WNS, screening visitors before cave tours, cleaning and decontaminating gear and clothing that has been in contact with bats or their habitats, and restricting access to caves when necessary to protect bats, other cave resources, and human safety [15–17]).

Many of these WNS preventative actions rely upon public participation. However, there is little to no information on visitors’ perceptions of or willingness to follow these actions aimed at slowing the spread of WNS. In this study, we applied the Theory of Planned Behavior (TPB), which maintains that attitudes, subjective norms, and perceived behavioral control shape an individual’s behavioral intentions, to determine underlying factors that explain U.S. national park visitors’ willingness to participate in a variety of WNS preventive strategies.

The TPB is a social psychology model that explains individuals’ intentions to enact behaviors and assumes that behaviors are enacted after planned, conscious, and deliberative thought [18]. According to the TPB, behavioral intention, defined as the strength of a person’s intention to perform a behavior, is the most important predictor of behavior [19]. Behavioral intention is predicted by three principal factors: a person’s attitude towards the behavior, subjective norms about the behavior, and perceived behavioral control regarding the performance of the behavior [20]. Attitudes are defined as the degree to which performance of the behavior is positively or negatively viewed, subjective norms are defined as the perceived social pressure to engage or not to engage in behavior, and perceived behavior control (PBC) is defined as a person’s perception of their ability to perform a given behavior [19]. It is assumed that attitudes and subjective norms have independent, direct relationships with behavioral intention, as a behavior can be deemed undesirable but socially acceptable. In contrast, PBC can also indirectly influence behavior if it moderates effects of attitudes or subjective norms on behavioral intent [19, 21–23]. (La Barbera & Ajzen 2021; Martinez and Lewis 2016; Ajzen 2019; Yzer 2007). For example, attitudes and subjective norms become less relevant in shaping intention when PBC is low because the action is not thought to be possible [24].

The TPB can be applied to affect behavior change [20], thus providing a starting point for organizations and agencies to develop wildlife conservation programs that rely upon human behavior. The TPB has frequently been used in the human dimensions of natural resource management to explain behaviors related to hunting [25, 26], recreation [27], and participation in conservation programs [28–30]. Additionally, there has been an increasing interest amongst bat researchers to utilize rigorous social science frameworks, like the TPB, to study and change human behaviors, as human activities are the primary cause of bat population declines around the world [31].

The objective of this study was to understand how visitors’ attitudes, subjective norms and perceived behavioral control related their behavioral intent to engage in five common WNS preventive strategies, including participating in educational programming, wearing clothes and shoes that have not been contaminated with *Pd*, walking over decontamination mats, and complying with cave closures. We predicted that park visitors would be willing to engage in all WNS preventive measures we addressed [32, 33]. We predicted attitudes, subjective norms, and PBC would all be determinants of behavioral intent. We also predicted that PBC would have a small moderating effect on the relationship between attitudes, subjective norms, and behavioral intent.

## Methods

### Study area

**Fig 1:**
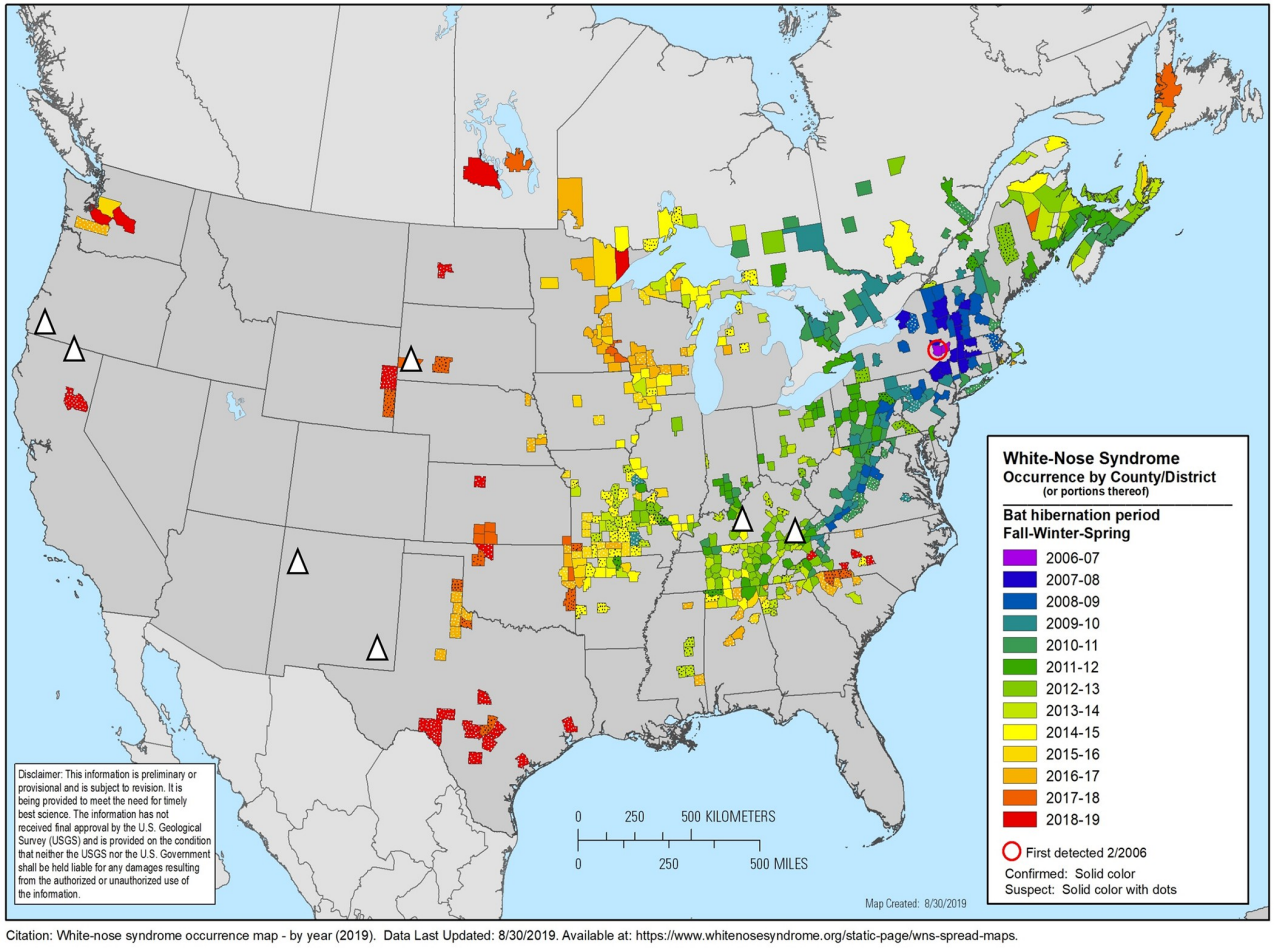
White-nose syndrome occurrence map at the time of our study. The triangles represent the locations of the national park areas we surveyed visitors were located. Wind and Jewel Cave (South Dakota) are located close to each other (approximately 47 km apart), so they are represented by a single triangle. Version from August 30, 2019, updated by the United States Fish and Wildlife Service. Available at https://www.whitenosesyndrome.org/where-is-wns.

The study area was comprised of eight U.S. national park units, including Oregon Caves National Monument and Preserve (Oregon), Lava Beds National Monument (California), Carlsbad Caverns National Park (New Mexico), El Malpais National Monument (New Mexico), Wind Cave National Park (South Dakota), Jewel Cave National Monument (South Dakota), Mammoth Cave National Park (Kentucky), and Cumberland Gap National Historic Park (Kentucky; Fig 1). These areas were chosen because they are spread across a wide geographical area, represent a diversity of cave ecosystems, and have varying WNS preventive measures. At the time of this study, WNS had been confirmed in Cumberland Gap, Mammoth Cave, Wind Cave, and Jewel Cave (Fig 1). White-nose syndrome had not been confirmed in Carlsbad Caverns, El Malpais, Oregon Caves, and Lava Beds (Fig 1). All parks in this study have hibernating species of bats that are vulnerable to WNS.

### Sampling design

We surveyed 18+ year old adult visitors at the eight national park units from June to August 2019. Our goal was to survey 175 visitors from each park, for a total of 1400 surveys. We utilized intercept sampling to recruit voluntary participants for a tablet-based, self-administered survey in or near the visitor center of each park based on the recommendations of park staff. Data were collected through the program iSurvey (www.harvestyourdata.com; Wellington, New Zealand). We had four tablets available for visitors to take the survey. When a tablet was available, we approached adult park visitors passing near our table and asked them to participate in this study. After visitors completed the survey, they were given a unique patch (i.e., collectible souvenir made from embroidered fabric) designed for the study as a reward for their time. On average, it took visitors 10 to 15 minutes to complete the survey. All research methods were reviewed and approved by the University of Tennessee’s Institutional Review Board (UTK IRB-19-05081-XM), the National Park Service’s Programmatic Review and Clearance Process (OMB Control No. 1024–0024), and science coordinators at each national park unit surveyed. The informed consent document was included on the first page of the survey. Visitors were instructed to read the informed consent and give verbal consent to the researchers or to continue the survey if they were okay with the information they were provided.

### Survey design

We conducted qualitative interviews with 15 key informants who worked closely with or for the NPS to better understand which WNS preventive actions were used by the parks so that we could create a targeted and robust quantitative survey [34]. The interviews were recorded, transcribed, and coded for potential items to include in the survey. We constructed five behavioral intent statements based on the most frequent cave management techniques and paired statements for attitudes, subjective norms, and perceived behavioral controls following the theory of planned behavior research protocols [20]. We pretested the survey by conducting cognitive interviews with 15 people within and outside of the fisheries and wildlife field to ensure that the questions were easy to understand and were conveyed the intended meaning [35]. We adapted question wording where appropriate before finalizing the survey instrument.

We used direct measures for behavioral intent, attitude, subjective norm, and PBC. The five WNS preventative actions addressed in this survey were 1) participating in educational programming on bat and cave conservation, 2) wearing clothes or shoes in caves that have not been contaminated with the fungus that causes WNS, 3) walking over decontamination mats before/after entering a cave, 4) complying with cave closures that last part of the year, and 5) complying with cave closures that last the entire year. Visitor behavioral intent, attitudes, subjective norms, and PBC towards the above WNS preventive actions were assessed using 5-point Likert-style scale questions (Tables 1–4). Behavioral intent was directly measured using a single statement for each of the five behavioral models (five-point scale from 1 = very unlikely to 5 = very likely; Table 1). Attitudes were directly measured using a single statement for each of the five behavioral models (five-point scale from 1 = very undesirable to 5 = very desirable; Table 2). Subjective norms were directly measured using three statements for each of five behavioral models (five-point scale from 1 = strongly disagree to 5 = strongly agree; Table 3). Perceived behavioral control was directly measured using three statements for each of five behavioral models (five-point scale from 1 = strongly disagree to 5 = strongly agree; Table 4). The survey was available in both English and Spanish. The Spanish survey was translated from the English survey by two native Spanish speakers, then back-translated to ensure consistency between the two questionnaires.

**Table 1:** Visitor behavioral intent about white-nose syndrome preventive actions used by the U.S. National Park Service, 2019.

Statement	Median	Mean^a^	SD	% Very Likely
If there are guided or recorded education programs or tours that focus on cave and bat conservation in national parks, how likely are you to participate?	Likely	4.00	0.95	32.7
If there are rules requiring visitors to wear clothes/shoes that have not been exposed to the fungus that causes white-nose syndrome, how likely are you to comply (even if it means changing clothes/shoes after entering the park)?	Very likely	4.56	0.81	69.0
If there are rules requiring visitors to walk over decontamination mats before and/or after entering a cave in a national park, how likely are you to comply?	Very likely	4.78	0.61	84.2
If there are cave closures that last part of the year in national parks to protect bats are, how likely are you to comply?	Very likely	4.71	0.67	78.6
If there are year-long cave closures in a national park to protect bats, how likely are you to comply?	Very likely	4.61	0.81	74.6

^a^ Ordered responses: 1 = very unlikely, 2 = unlikely; 3 = neither unlikely nor likely, 4 = likely, 5 = very likely

**Table 2:** Visitor attitudes about white-nose syndrome preventive actions used by the U.S. National Park Service, 2019.

Statement	Median	Mean^a^	SD	% Very Desirable
For me, participating in live or recorded education programs and tours that focus on cave and bat conservation is:	Desirable	4.04	0.91	36.0
For me, being required to wear clothes/shoes that have not been exposed to the fungus that causes white-nose syndrome when entering a cave in a national park (even if it means changing clothes/shoes after entering the park) is:	Very desirable	4.33	0.91	54.9
For me, being required to walk over decontamination mats that remove the fungus that causes white-nose syndrome from my shoes before and/or after entering a cave in a national park is:	Very desirable	4.53	0.75	65.6
For me, complying with cave closures that last part of the year in national parks to protect bats is:	Very desirable	4.45	0.86	62.1
For me, complying with cave closures that last all year in national parks to protect bats is:	Very desirable	4.29	1.01	57.5

^a^ Ordered responses: 1 = very undesirable, 2 = undesirable; 3 = neither undesirable nor desirable, 4 = desirable, 5 = very desirable

**Table 3:** Visitor subjective norm responses to the statement “I am more likely to do [white-nose syndrome preventative] action if [a ranger tells me to do it, the group I am traveling with is doing it, other visitors are doing it],” 2019.

White-nose Syndrome Preventive Action	Median	Mean^a^	SD	% Strongly Agree
Educational Programming		---	---	
Information about it is written on a sign	Agree	4.10	0.88	35.5
A ranger tells me about it	Agree	4.15	0.86	39.2
Traveling group is doing it	Agree	4.00	0.95	34.0
Other visitors are doing it	Agree	3.65	1.08	25.2
Wearing clean clothes/shoes		---	---	
Information about it is written on a sign	Strongly agree	4.36	0.78	50.9
A ranger tells me about it	Strongly agree	4.54	0.69	62.7
Traveling group is doing it	Agree	4.07	1.02	43.0
Other visitors are doing it	Agree	3.97	1.08	40.5
Walking over decontamination mats		---	---	
Information about it is written on a sign	Strongly agree	4.50	0.71	59.7
A ranger tells me about it	Strongly agree	4.63	0.63	68.8
Traveling group is doing it	Agree	4.17	1.01	48.4
Other visitors are doing it	Agree	4.09	1.05	46.0
Complying with partial cave closures		---	---	
Information about it is written on a sign	Strongly agree	4.51	0.71	60.7
A ranger tells me about it	Strongly agree	4.59	0.66	66.7
Traveling group is doing it	Agree	4.12	1.03	46.4
Other visitors are doing it	Agree	4.02	1.08	43.6
Complying with year-long cave closures		---	---	
Information about it is written on a sign	Strongly agree	4.49	0.78	61.0
A ranger tells me about it	Strongly agree	4.56	0.73	66.4
Traveling group is doing it	Agree	4.06	1.07	45.2
Other visitors are doing it	Agree	3.97	1.11	41.8

^a^ Ordered responses: 1 = strongly disagree, 2 = disagree; 3 = neither disagree nor agree, 4 = agree, 5 = strongly agree

**Table 4:** Visitor perceived behavioral control responses to the statement “Whether or not I participate/comply with [WNS preventative action] is [completely up to me, influenced by my resources, influenced by my awareness],” 2019.

White-nose Syndrome Preventative Action	Median	Mean^a^	SD	% Strongly Agree
Educational Programming	---	---	---	---
Completely up to me	Agree	4.11	1.05	44.6
Influenced by resources	Agree	3.75	1.08	25.1
Influenced by knowledge	Agree	4.13	0.88	37.2
Wearing clean clothes/shoes	---	---	---	---
Completely up to me	Agree	3.49	1.45	34.2
Influenced by resources	Agree	3.43	1.27	22.0
Influenced by knowledge	Agree	4.15	1.08	46.6
Walking over decontamination mats	---	---	---	---
Completely up to me	Agree	3.19	1.57	30.7
Influenced by resources	Neither	2.84	1.45	17.7
Influenced by knowledge	Agree	3.95	1.25	43.2
Complying with partial cave closures	---	---	---	---
Completely up to me	Agree	3.22	1.55	30.8
Influenced by resources	Neither	2.85	1.43	17.0
Influenced by knowledge	Agree	3.96	1.26	44.0
Complying with year-long cave closures	---	---	---	---
Completely up to me	Neither	3.17	1.57	30.3
Influenced by resources	Neither	2.81	1.42	16.1
Influenced by knowledge	Agree	3.94	1.27	43.7

^a^ Ordered responses: 1 = strongly disagree, 2 = disagree; 3 = neither disagree nor agree, 4 = agree, 5 = strongly agree

## Statistical analyses

**Fig 2:**
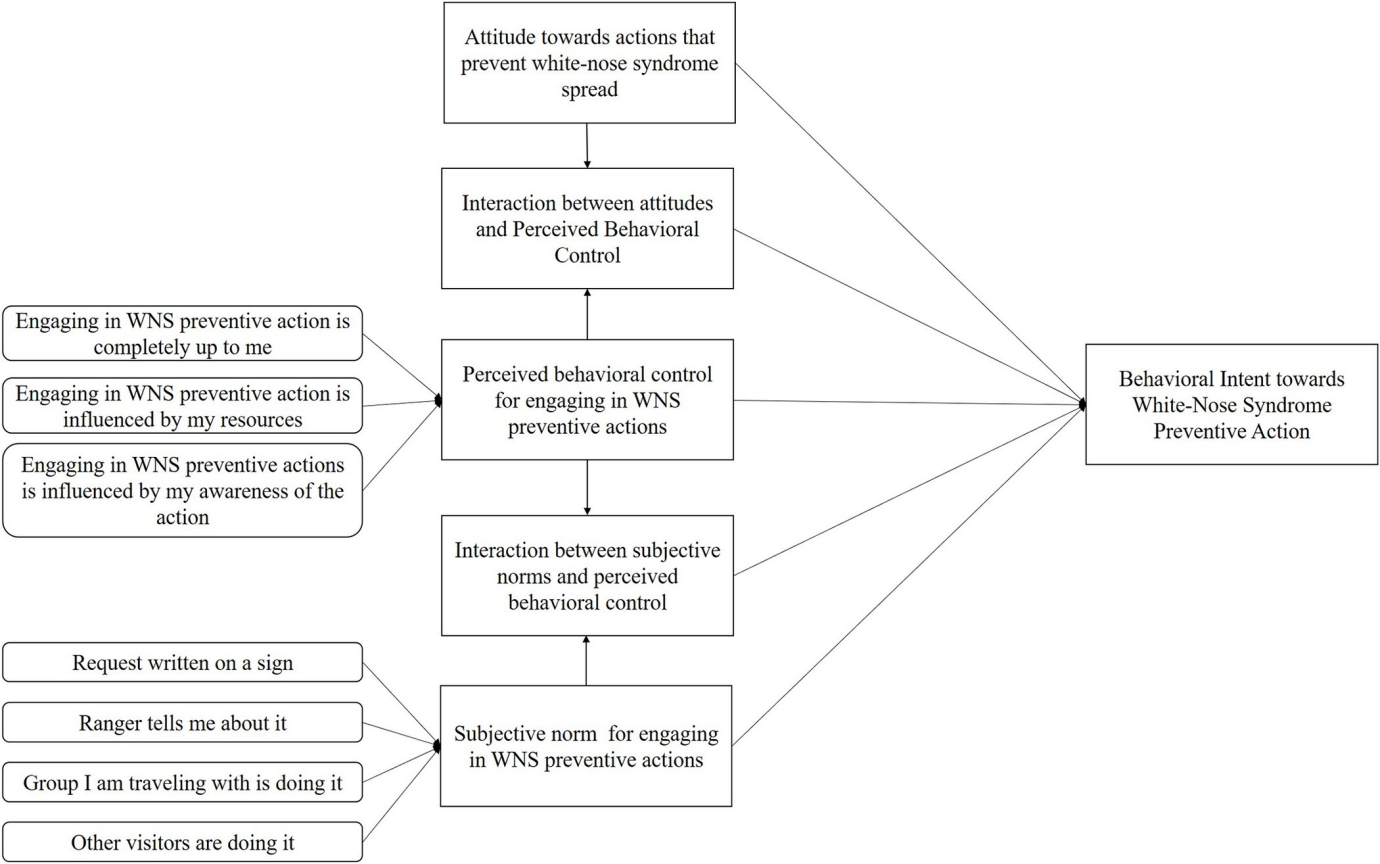
Example of the theory of planned behavior template used for each of the five white-nose syndrome preventive strategies used in U.S. national parks.

We assessed non-response error by comparing sociodemographic data from our survey with frequencies available from a National Park Service report titled “Linking the 2010 Census to National Park Visitors” [36]. Gender data were not available from this report and were not considered in this analysis. We used Cronbach’s alpha to assess reliability of the statements used to measure subjective norms and perceived behavioral control [37] and confirmatory factor analysis to measure these statements’ discriminant and convergent validity within and among our constructs that included multi-item indicators (i.e., subjective norms, perceived behavioral control; S1–S5 Figs) [38]. All independent variables were mean centered prior to analysis [39]. The mean-centered variables were used to generate interaction terms [39] between attitudes and perceived behavioral control and between subjective norms and perceived behavioral control to understand if perceived behavioral control had a moderating effect on these two variables [21, 40]. We employed Structural Equation Modeling (SEM) with maximum likelihood estimation to determine the relationships between behavioral intent (response variable) and the calculated direct measures of attitude, subjective norm, PBC, and the interaction terms (explanatory variables) (Fig 2). We chose SEM because it is a more flexible tool than regression modeling. All analyses were conducted in SPSS 27 (IBM SPSS Statistics for Windows, Version 27.0, New York: IBM Corp) and Amos 27 Graphics (IBM SPSS AMOS for Windows, Version 27.0, Chicago: IBM Corp).

## Results

### Response, demographics, and visitor characteristics

We collected 1365 completed surveys. Response rates varied between parks, and overall, non-compliance averaged around 49%. We defined non-compliance as visitors who declined to take the survey after being spoken to by the researchers. We reached our survey goal at every park except El Malpais (n = 125). Additionally, we inadvertently surveyed 15 additional people at Mammoth Cave (n = 190). The sample approximated typical national park visitors [36], minimizing the likelihood of non-response bias. Respondents were 58% female, 86% white, and averaged 45 ± 14 (Mean ± SD) years old. Most respondents had earned a bachelor’s degree (n = 409; 30%) or more than a bachelor’s degree (n = 490; 35%). Most respondents were planning on touring the caves in the national park they were visiting (n = 659; 48%) or had already toured the caves (n = 421; 31%). Most respondents (n = 791; 58%) indicated that it was their first time visiting the national park in which they took the survey, and roughly half (n = 636; 53%) of the respondents noted that they have visited caves in other national parks.

### Theory of planned behavior analyses

#### Behavioral intent

Respondents were likely to participate in guided or recorded educational programs or tours that focused on bat and cave conservation. Respondents were very likely to wear clothes/shoes that have not been exposed to the fungus that causes WNS (even if it meant changing their clothes/shoes after they entered the park), to use decontamination mats before and/or after entering a cave, to comply with cave closures that lasted part of the year, and to comply with cave closures that lasted all year (Table 1). For each of these actions, only a small percentage (1.60% to 7.90%) of respondents were unlikely to participate (Table 2).

#### Attitudes

Respondents had positive attitudes towards all WNS preventive actions addressed in this study, with respondents expressing the most positive attitudes towards walking over decontamination mats before and/or after entering the cave (65.6% responded very desirable). On average, respondents felt that participating in educational programming was desirable and engaging in the other WNS preventive measures was very desirable. For each of these actions, only a small percentage (1.90% to 7.30%) of respondents felt they were undesirable (Table 2).

#### Subjective norms

Visitors agreed that all subjective norms addressed in this study positively influenced them to participate or comply with the WNS preventative actions (Table 3). Respondents were most influenced by rangers discussing these measures with them (39.2–68.8% strongly agreed), followed by signage (35.5–61.0% strongly agreed), the actions of respondents’ traveling group (34.0–48.4% strongly agreed), and actions of visitors outside of the respondent’s group (25.2–46.0% strongly agreed). The four subjective norm statements exhibited a high Cronbach’s alpha in every behavior (0.79–0.81), indicating adequate construct validity and reliability.

#### Perceived behavioral control

Visitors agreed that their participation in WNS preventative actions was influenced by their prior awareness of the preventive actions. Visitors also stated that participation in educational programming and wearing clothes or shoes in caves that have not been contaminated with the fungus that causes WNS was completely up to them and was influenced by their resources (Table 4). However, visitors neither agreed nor disagreed that walking over decontamination mats and complying with cave closures was completely up to them or influenced by their resources. The three perceived behavioral control statements factored into have an acceptable Cronbach’s α between 0.69 and 0.70, indicating adequate construct validity and reliability [41].

#### Theory of planned behavior model results

Overall, the five models explained 21% - 39% of the variance of the dependent variables (Table 5; S1–S5 Figs). The test for goodness-of-fit was acceptable for all models based on the following measures [42]: RMSEA (0.048–0.068), CFI (0.962–0.974), GFI (0.971–0.983), and SRMR (0.031–0.050; S1 Table). The positive relationship between attitudes (β = 0.35–0.56) and behavioral intent had the largest effect size for all WNS preventive actions according to the SEM standardized results (Table 5). Subjective norms had the second largest positive effect on behavioral intent (β = 0.12–0.27). Perceived behavioral controls only had a significant influence on behavioral intent for complying with year-long cave closures (Table 5). The interaction between attitudes and PBC had a significant influence on behavioral intent for complying with partial and year-long closures, and the interaction between subjective norms and PBC had a significant influence on behavioral intent for participating in educational programming, using decontamination mats, and complying with partial cave closures (Table 5).

**Table 5:** Structural equation modeling standardized outputs explaining how likely national park visitors were to participate or comply in white-nose syndrome preventive actions using the three components of the theory of planned behavior (attitudes, subjective norms [SN], perceived behavioral control [PBC]) and two interaction terms (attitude*PBC; SN*PBC).

Predictor	Educational Programming (r^2^ = 0.39)	Wearing Clean Clothes/Shoes (r^2^ = 0.31)	Decontamination Mats (r^2^ = 0.21)	Partial Closure (r^2^ = 0.28)	Year-long Closure (r^2^ = 0.35)
**Main Analyses**	β	β	β	β	β
Attitude	0.56*	0.46*	0.35*	0.38*	0.43*
SN	0.12*	0.18*	0.17*	0.26*	0.27*
PBC	0.04	-0.04	-0.04	-0.05	-0.09*
**Interaction**					
Attitude*PBC	0.00	0.02	0.05*	0.08*	0.11*
SN*PBC	-0.06*	-0.02	-0.09*	-0.07*	-0.02

*p < 0.05

## Discussion

In national parks, the successful implementation of WNS preventive actions relies on the support and participation of the hundreds of thousands of visitors who tour caves annually. It is therefore encouraging that most visitors who participated in this study were willing or very willing to participate in or comply with five commonly used WNS prevention actions, even if those actions restricted their access to NPS cave resources. Visitors had positive attitudes towards these actions, and their likelihood of engaging in these actions was positively influenced by signage, park staff, and other visitors. In alignment with our predictions, we found that attitudes and subjective norms were both determinants of visitors’ behavioral intent; however, PBC was only a determinant for compliance with cave closures. We also found PBC had a small moderating effect on attitudes and subjective norms’ relationship with behavioral intent, although the direction and significance of this effect varied between WNS preventive behaviors.

Visitors expressed positive attitudes towards every WNS preventive action addressed in this study. This result is not unique, as a study on visitor support for bat management in historic buildings in Great Smoky Mountain National Park found that visitors were very likely to support management actions that benefited bat conservation, even if these strategies restricted visitor access to certain historical sites [32]. Consistent with previous research [e.g., 25, 27, 28, 43], attitudes towards a specific action had the greatest positive effect on behavioral intent. It will be important for parks to develop or continue to use persuasive communications to maintain visitors’ positive attitudes towards these actions. Interviews with park staff and observations by the researchers during this study revealed that most visitors support or participate in WNS preventive actions when paired with educational materials or other means of explaining the reasoning for these measures [34]. Additionally, this communication and messaging should aim to maintain park visitors’ positive attitudes towards bats [33] and directly explain how these measures protect vulnerable bat populations, as previous studies indicated that attitudes towards bats had one of the greatest effects on support for bat management [32, 34].

Subjective norms were also identified as an important, positive predictor of behavioral intent for WNS preventive actions. Visitors agreed or strongly agreed that they were more likely to engage in WNS preventive actions if signage and park staff informed them about the action or their traveling group and other visitors were engaging in those actions. Unsurprisingly, visitors were more likely to be influenced by park signage and staff compared to visitors they were travelling with or other visitors, as park staff and official messaging from the parks are likely viewed with more authority than fellow visitors. Many studies have examined how to craft persuasive messaging to promote safe behaviors in national parks [e.g., 44–46]. Our study highlights the importance of considering injunctive norms (i.e., perception of others approval or disproval of an action) compared to descriptive norms (i.e., perception of how others behave) when designing messages [44]. Based on our results, visitors may be more influenced by messaging communicated through signage and park staff about what is acceptable behavior in the parks to protect vulnerable bat populations (e.g., walking over decontamination mats, not entering caves) compared to messaging or observations focused on what other park visitors are doing.

The value of creating an effective communication campaign about WNS preventive actions is especially important in parks with decentralized cave systems and ones without guided cave tours. Some research in national parks suggests direct, in-person interactions between park visitors and staff is more likely to lead to attitude and behavior change [47], but that type of interaction is not possible in all situations. For example, Lava Beds National Monument has over 20 dispersed caves that are open to visitors to explore on their own. It is not a guarantee that visitors will first stop at the visitor center, and it is not possible for park staff to be present at the entrance of every cave. To address this issue, park staff provide information about WNS preventive actions at the fee booths when they arrive at the park, instruct visitors to pick up a caving permit at the visitor center if they intend to explore the caves, provide a variety of educational programs about bats, and post signs at cave entrances to remind visitors of these requirements [48]. This combined approach enabled the park to engage an estimated 98% of visitors in 2018 in education and prevention of WNS through interpretation, cave permits and closures, and a shoe cleaning station, which improved efficiencies and compliance with these measures (Smith unpublished work).

Perceived behavioral control was only a significant predictor of behavioral intent for complying with year-long cave closures. However, compared to attitudes and subjective norms, it was the least influential component for this WNS preventive action. Previous studies have found that PBC is often a less influential factor when people’s decisions are under volitional control, meaning outside factors do not limit their intent when considering whether or not to perform a behavior [25, 49]. Although visitors may consider aspects of PBC (i.e., ability, resources, knowledge) when deciding on whether to engage WNS preventive measures, visitors did not perceive that these factors substantially affected their intent to participate in these actions. This result may be explained by NPS’s efforts to remove barriers for visitor participation in WNS preventive actions. For example, some parks had the decontamination mats right at the entrance or exit of the cave, so visitors would not have to go out of their way to decontaminate their shoes.

Despite the limited direct effect of PBC on behavioral intent, it is important for managers to consider this component when developing or implementing new WNS preventive actions. Park response to WNS varies between parks, as the natural resources, infrastructure, and layout of each park is different [34]. Thus, there is often not a one-size-fits-all approach to implementing WNS preventive measures, and visitors may respond differently to these measures based on the park they are visiting. For example, some parks had decontamination mats at the entrance or exit of the caves, whereas others only had these mats at certain locations (e.g., visitor center, trailheads). It will be important to understand if these different mat placements influence visitors’ PBC, behavioral intentions, and actual behaviors. Additionally, interactions between PBC, attitudes, and subjective norms had small but significant effects on behavioral intent for most of the preventive strategies. This result suggests PBC had a moderating effect on attitudes’ and subjective norms’ relationship with behavioral intention. Previous research has found that attitudes and subjective norms can become less relevant in shaping intention when PBC is low because the action is not thought to be possible [21, 24]. It will be important for parks to monitor visitors’ engagement with WNS preventive measures, understand potential barriers to engaging in these actions, and implement communication and management strategies to increase compliance when necessary.

### Limitations and future research

There are several limitations and avenues for future research. First, it is possible some respondents may have inaccurately stated their behavioral intent and attitudes towards participation in WNS prevention actions due to social desirability bias of being in a national park where these actions are encouraged. It is also possible that there was a ceiling effect due to the high means and limited variability for visitor support of some actions. Second, in order to reduce respondent burden, we only used single-item indicators for behavioral intent and attitudes. This may have created a simplified picture of respondents’ perceptions of actions used to prevent WNS. Third, this approach examined behavioral intent and did not assess actual visitor participation in WNS preventive actions. While intention is seen as the immediate antecedent of behavior, it will be important for parks to assess actual visitor behavior through observational studies to better understand visitor participation rates in WNS preventive strategies [45].

Lastly, although a large body of TPB research has centered on examining the drivers behind a suite of visitor behaviors (e.g., “leave no trace” [46, 50], using bear spray [43], safely viewing wildlife [45]), it is important to recognize the limitations of this framework. Previous research has cast doubt on the relationships between components of the TPB (e.g., PBC and behaviors, intentions and behaviors), identified inconsistencies in how components of the TPB are operationalized, and highlighted how the rational actor assumption ignores the role that emotions and other variables play on influencing human behavior [51]. Our study provides a good starting point for understanding visitors’ willingness to engage in WNS preventive actions and the factors that influence these behaviors, but future research should consider and try to integrate how other variables not included in this study (e.g., emotions [52], risk perceptions [53], values [54], trust in government [55]) influence both behavioral intentions and actual behaviors.

## Conclusions

WNS poses a serious threat to the health of North American bat populations and current ecosystem structure and function. Many organizations working to conserve bat populations, including The National Park Service, have created and implemented a variety of preventive actions to slow the spread of WNS and minimize anthropogenic disturbance of vulnerable bat populations. We found that national park visitors in our sample were likely or very likely to engage in all WNS preventive actions addressed in this study, including participating in educational programming on bat conservation, wearing clothes and shoes that have not been exposed to *Pd*, walking over decontamination mats before and/or after touring a cave, and complying with partial or year-long cave closures. Attitudes and subjective norms were key influences on visitors’ behavioral intent towards these actions. We suggest that parks develop or continue to use multiple communications strategies (e.g., signage, park staff messaging, tours) to convey the importance of these measures for protecting bat populations. Messaging should also emphasize the need for visitor participation and compliance for the success of the actions. Additionally, we recommend that parks monitor for barriers to engaging in new WNS preventive strategies and use appropriate communications and management strategies to minimize these barriers when necessary. As cave management actions continue to change over the coming decade, evaluating visitors’ perceptions and participation in specific disease prevention strategies will be crucial for achieving management goals focused on enhancing recreation and conservation in environments shared by humans and bats.

## Supporting information

S1 Dataset Complete dataset used in analysis. (XLSX)

S1 Table Goodness of fit statistics for all models. (PDF)

S1 Fig Structural equation model for visitor willingness to engage in educational programming. (PDF)

S2 Fig Structural equation model for visitor willingness to wear clothes and shoes that have not been exposed to *Pd*. (PDF)

S3 Fig Structural equation model for visitor willingness to walk over decontamination mats before and/or after entering a cave in a national park. (PDF)

S4 Fig Structural equation model for visitor willingness to comply with cave closures that last part of the year. (PDF)

S5 Fig Structural equation model for visitor willingness to comply with year long cave closures. (PDF)
