# Supplementary material for: U.S. National Park visitor perceptions and behavioral intentions towards actions to prevent white-nose syndrome
Source: PLoS One. 2022 Nov 23;17(11):e0278024. doi: 10.1371/journal.pone.0278024 (PMC9683549; doi:10.1371/journal.pone.0278024)
Supplement: S1 Table — (PDF) [file pone.0278024.s002.pdf]

S1 Table. Goodness of fit statistics for all five models utilizing the Theory of Planned Behavior to determine influences on visitor compliance with or participation in white-nose syndrome preventive measures.

	RMSEA	CFI	GFI	SRMR
Participating in educational programs	0.048	0.974	0.983	0.031
Wearing clothes/shoes that have not been contaminated by the fungus	0.064	0.962	0.972	0.041
Walking over decontamination mats	0.055	0.973	0.979	0.041
Complying with partial cave closures	0.064	0.965	0.974	0.050
Complying with year-long cave closures	0.068	0.962	0.971	0.051

*We used the following criteria to determine acceptable goodness-of-fit statistics (Hooper et al. 2008): RMSEA < 0.08; CFI > 0.90; GFI > 0.90; SRMR <0.08.
