# Supplementary material for: U.S. National Park visitor perceptions and behavioral intentions towards actions to prevent white-nose syndrome
Source: PLoS One. 2022 Nov 23;17(11):e0278024. doi: 10.1371/journal.pone.0278024 (PMC9683549; doi:10.1371/journal.pone.0278024)
Supplement: S3 Fig — (PDF) [file pone.0278024.s005.pdf]

S3 Fig. Full structural equation model using the Theory of Planned Behavior regarding national park visitors' willingness to walk over decontamination mats before and/or after entering a cave in a national park.

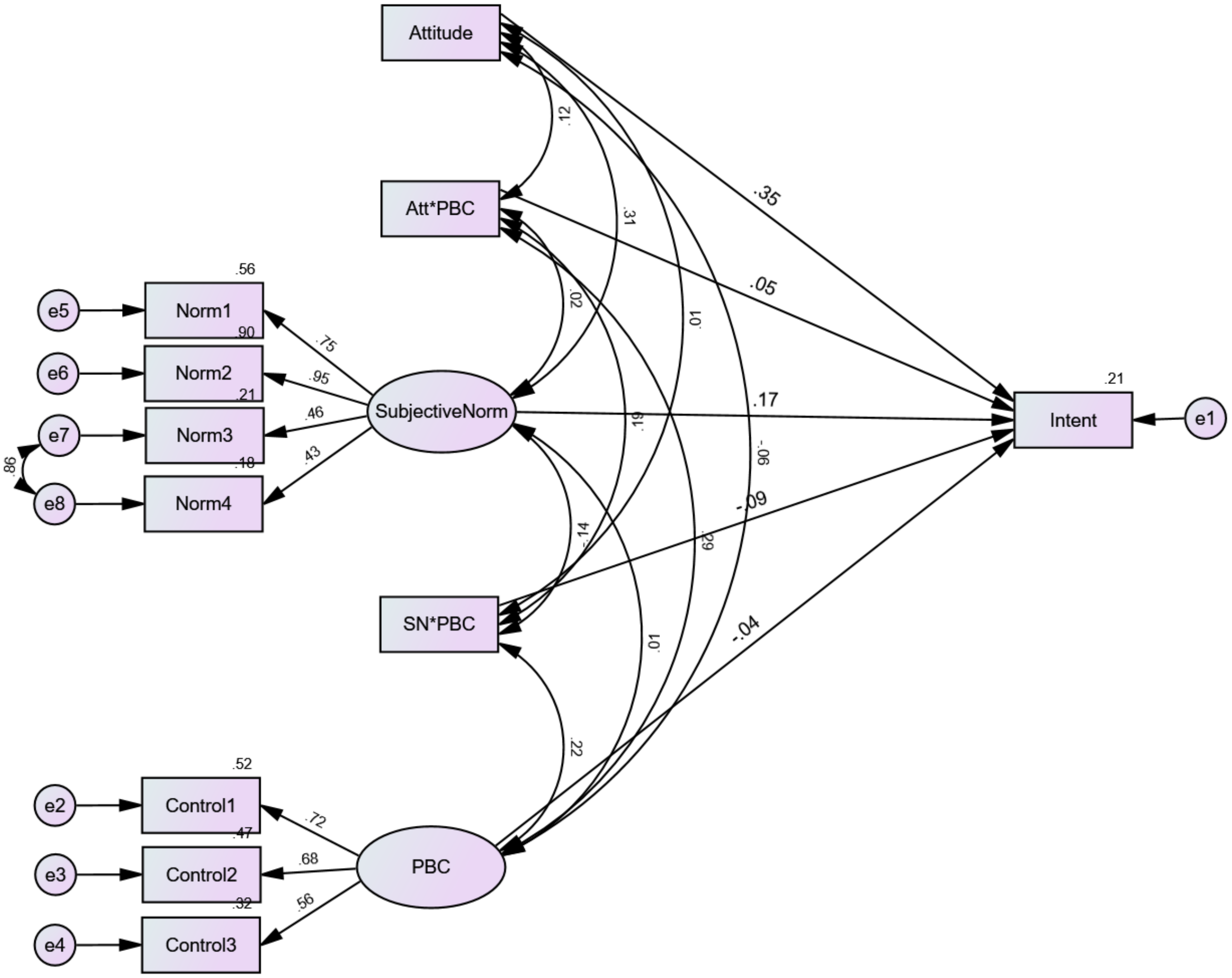

Variable Definitions:

- Norm1: I am more likely to walk over decontamination mats if information about it is written on a sign
- Norm2: I am more likely to walk over decontamination mats if a ranger tells me about it
- Norm3: I am more likely to walk over decontamination mats if my traveling group is doing it
- Norm4: I am more likely to walk over decontamination mats if other visitors are doing it
- Control1: Whether or not I walk over decontamination mats is completely up to me
- Control2: Whether or not I walk over decontamination mats is influenced by my resources
- Control3: Whether or not I walk over decontamination mats is influenced by my prior knowledge
