# Supplementary material for: U.S. National Park visitor perceptions and behavioral intentions towards actions to prevent white-nose syndrome
Source: PLoS One. 2022 Nov 23;17(11):e0278024. doi: 10.1371/journal.pone.0278024 (PMC9683549; doi:10.1371/journal.pone.0278024)
Supplement: S4 Fig — (PDF) [file pone.0278024.s006.pdf]

S4 Fig. Full structural equation model using the Theory of Planned Behavior regarding national park visitors' willingness to comply with comply with cave closures that last part of the year in national parks.

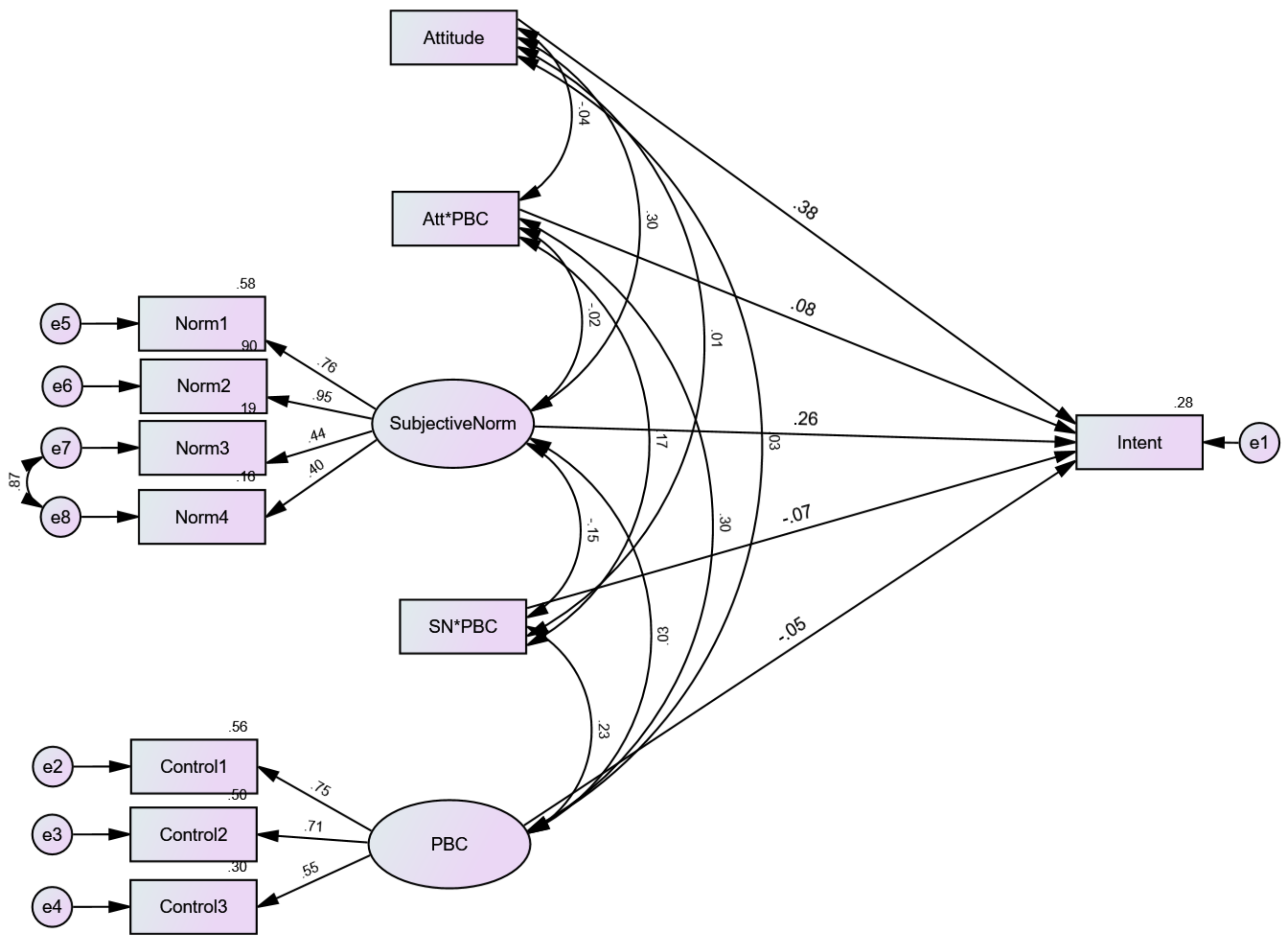

Variable Definitions:

- Norm1: I am more likely to comply with cave closures that last part of the year if information about it is written on a sign
- Norm2: I am more likely to comply with cave closures that last part of the year if a ranger tells me about it
- Norm3: I am more likely to comply with cave closures that last part of the year if my traveling group is doing it
- Norm4: I am more likely to comply with cave closures that last part of the year if other visitors are doing it
- Control1: Whether or not I comply with cave closures that last part of the year is completely up to me
- Control2: Whether or not I comply with cave closures that last part of the year is influenced by my resources
- Control3: Whether or not I comply with cave closures that last part of the year is influenced by my prior knowledge
